# Assessing the Estimands and Estimates of Hospitalization Rates in Health Economics and Clinical Medicine

**DOI:** 10.1002/hec.70117

**Published:** 2026-06-18

**Authors:** Aditya Jain, Gil Peled, Filip Obradović, Federico Crippa, Yeshaya Nussbaum, Michael Gmeiner, Daniela P. Ladner, Charles F. Manski

**Affiliations:** ^1^ Department of Economics Northwestern University Evanston Illinois USA; ^2^ Department of Economics London School of Economics London UK; ^3^ Northwestern University Transplant Outcomes Research Collaborative (NUTORC) Comprehensive Transplant Center (CTC) Feinberg School of Medicine Northwestern University Chicago Illinois USA; ^4^ Division of Transplantation, Department of Surgery Northwestern Medicine Chicago Illinois USA; ^5^ Institute for Policy Research Northwestern University Evanston Illinois USA

**Keywords:** healthcare, health data, hospitalization, household production, medicare

## Abstract

Even though data on hospital admissions are widely used in health research, hospitalization‐related estimands measured using these data are not always clearly conceptualized. Consequently, estimators of these quantities can have unclear rationales and undesirable properties. We evaluate three “rate” estimators for measuring hospitalization‐related estimands. Using the Grossman human capital model, we motivate the importance of measuring healthy time. We show that an upper bound on healthy time can be calculated using lengths of hospital stay without assumptions about health status outside the hospital. We illustrate the empirical value of these bounds. Next, we find that an admission rate conventionally used in clinical research is a patient follow‐up time weighted average that lacks a clear basis for the weights. We propose an alternative estimator with more desirable properties and weaker assumptions. We assess its performance using a model of hospital admissions and death. Finally, we evaluate the Centers for Medicare and Medicaid Services (CMS) use of risk‐standardized readmission rates to penalize hospitals by showing that risk‐standardized rates can be sensitive to patient case mix, potentially leading to hospital rankings that do not reflect hospital quality. We propose treating hospital specific intercepts in the CMS risk‐standardization model as a measure of quality.

## 1 Introduction

Health, administrative, and financial datasets on hospital admissions and stays offer a rich source of information for answering questions that could improve clinical practice and public health policy. Researchers in applied health measure various hospitalization‐related rates. For example, in clinical research hospital admission and readmission rates are used to understand disease progression. Readmission rates are tied to national quality metrics and reimbursement. Bed occupancy rates are used to assess hospital performance. Health economists may use hospitalization data to understand the economic risk of adverse health shocks and measure utilization‐based time allocation outcomes. The costs of providing care, including those for inpatient stays, are used to measure the burden of disease on healthcare system resources. Costs borne by patients estimate the financial burden on individuals.

In practice, algebraic measures related to hospitalization have been computed without clearly conceptualizing the estimand of interest. Consider the hospital admission rate conventionally measured in clinical research. Written formally, this is

$$\begin{array}{r} \frac{365\sum_{i}n_{i}}{\sum_{i}d_{i}}, \end{array}$$

where the summations are over all patients i in a patient cohort observed in a specified time period, $n_{i}$ denotes the number of hospital admissions for patient i and $d_{i}$ is the number of days that patient i is observed. Section 3 will show that this rate is equivalent to a weighted average of individual hospital admission rates, with weights proportional to the time a patient is observed in the data. Metcalfe et al. (2003) note that studies heuristically use this definition “to counter” variable follow‐up lengths in the patient cohort. We will argue in Section 3.2 that the substantive rationale for using this weighting scheme when defining the admission rate is still unclear. We show how an alternative estimator can better measure the estimand of interest to clinical researchers.

The lack of satisfactory appraisals for hospitalization rates reported in medical and public health research motivates us to describe and compare useful rates for measuring important hospitalization‐related quantities in health economics and clinical medicine subspecialities. The same name sometimes refers to rates for measuring different quantities. For instance, “readmission rates” used in clinical research on disease progression and hospital performance assessment are defined differently. This makes it especially important to discuss the reasoning and motivation behind defining an estimator and what estimand those estimators are attempting to measure.

We evaluate three distinct hospitalization‐related estimands across different fields of health research. In each case, we follow a parallel structure: we articulate the quantity of interest, assess the mathematical or conceptual limitations of conventionally used estimators, and propose an alternative. In Section 2, we discuss healthy time as an estimand of interest in health economic studies of health capital and production. We propose a hospitalization rate that estimates an upper bound on healthy time. We illustrate how knowledge of this upper bound can correct upward biases in estimations of QALYs and health capital.

Section 3 discusses a common definition of admission rates used to study disease progression in clinical research. We propose an alternative admission rate that appropriately counts patients who are observed for varying amounts of time. Section 4 evaluates the Centers for Medicare and Medicaid Services (CMS) use of risk‐standardized 30‐day readmission rates in the Hospital Readmissions Reduction Program (HRRP). We highlight potential issues with using standardized readmission ratios to assess hospital performance and calculate payment reductions. As an alternative, we suggest that hospital‐specific intercepts in the CMS model be interpreted as measures of unobserved quality and used to rank hospitals.

To our knowledge, no existing research in clinical medicine or health economics has reviewed or interpreted the definitions of alternative hospitalization rates for hospital utilization data. In clinical medicine, the need for “adjustment” of individual admission rates when the follow‐up times differ between groups of patients is a recognized problem (Metcalfe et al. 2003). Our proposed admission rate aggregates patient‐specific rates rather than using a cohort‐wide rate. A second contribution is our use of hospital admissions data to estimate an upper bound on the distribution of individual healthy time. This responds to the need cited by Burns and Mullahy (2016) for “downstream research” on measurement of health status when individuals are not in contact with the healthcare system. Our third contribution builds on production theory in applied econometrics to recognize that within the CMS risk‐standardized model, ranking hospitals through their specific intercepts could provide a sound estimator of their unobserved quality.

## 2 Health Economics: Health Capital & Production

This section evaluates the measurement of “healthy time” in health economics. Section 2.1 formalizes healthy time as an estimand using the Grossman human capital framework. Section 2.2 proposes an estimator for an upper bound on healthy time using data on the duration of hospital admissions. Section 2.3 illustrates the value of this bound using an example of health capital estimation.

### 2.1 Healthy Time in the Grossman Model

Much empirical health economics research depends on measuring individuals' health status (Mullahy 2016). Burns and Mullahy (2016) call time‐denominated measures of health as characterizing and measuring “healthy time.” The Grossman (1972) human capital model of the demand for health was the first to formalize the importance of healthy time for individuals. Grossman's work spawned several theoretical and empirical extensions of his framework (Grossman 1982; Rosenzweig and Schultz 1983). For our purposes, Grossman's human capital model is sufficient to motivate the importance of healthy time as an outcome in empirical research.

Grossman's intertemporal utility function for consumer i in a given period t, say a year, is given by

$$\begin{array}{r} U_{it}=U\left(\phi_{it}H_{it},Z_{it}\right), \end{array}$$

where $H_{it}$ is the health capital stock in year t, $Z_{it}$ denotes the consumption of another commodity (such as leisure) in this year, and $\phi_{it}$ is the flow of health services in year t per unit of health stock $H_{it}$. Grossman (2000) assumes $\phi_{it}H_{it}$ to equal healthy time in the year by stating that the health stock provides no services besides healthy time. Consumers produce health $H_{it}$ and other commodities through production functions that take market goods (medical care utilization for health) and allocated time as inputs.

Healthy time may not be directly observable; however, its empirical bounds can be determined by decomposing an individual's time budget. Let Ω=365 be the number of days available in year t. The time budget Ω can be broken into its mutually exclusive component shares $TL_{it}+TH_{it}+T_{it}=\Omega ,$ where TH is time allocated toward investment in health H (such as time spent exercising), TL is time lost to illness or injury, and T is time devoted to work or the production of other commodities such as leisure activities.^1^ Healthy time is the total number of days neither ill nor injured in the year, that is, $h_{it}=\Omega -TL_{it}$. The quantity $h_{it}=\phi_{it}H_{it}$ also represents healthy time in year t, when the health stock is assumed to yield only healthy time as a service. In this context, $\phi_{it}$ represents how productively consumer i can generate healthy time from a unit stock of health in year t. Healthy time $h_{it}$ is a consumption commodity that directly enters a person's utility function. It is intuitive that people value healthy time and that time spent ill is a source of disutility (Ganguli 2024).

### 2.2 Upper Bounds on the Distribution of Individual Healthy Time

A hospital‐utilization‐based measure of healthy time has to contend with the issue that time spent outside the hospital may not necessarily be time spent healthy, but time spent ill. While discussing utilization‐based measures of health outcomes, Mullahy (2016) notes that the premise that “a given day within the accounting period has a positive value if the individual is alive and not in contact with the healthcare system on that day … merits scrutiny in some contexts.” A day when an individual is not in contact with the healthcare system has a positive value, according to Grossman's model, if the patient is not ill and can thus allocate that time toward work, improving their health, or other activities that provide utility. Patient‐centered outcomes of health status such as *Contact Days* (The ESCAPE Investigators and ESCAPE Study Coordinators 2005) and *Days Alive Out of Hospital* (Medicare Payment Advisory Committee (MedPAC) 2015; Meza et al. 2024) are operationalized as time spent not in contact with the healthcare system. Nevertheless, healthcare systems across OECD countries have struggled with long waiting times for various health services (OECD 2020, 2025). A patient waiting to receive care is likely to be unhealthy for at least a part of their waiting period.

Since time spent outside the hospital is not necessarily healthy, we propose the following rate as an upper bound on healthy time for a patient i in year t. In a cohort for which a researcher has inpatient admission data, let $d_{it}$ denote the follow‐up time (time that a patient is observed by the researcher, such as the time that they are enrolled in health insurance) in days for patient i in year t. Let $l_{it}$ denote the total number of days during the follow‐up period that patient i is hospitalized. Using the $\Omega -{TL}_{it}$ definition of healthy time, define the following rate for patient i in year t with 365 days,

$$\begin{array}{r} {h_{U}}_{it}=\frac{\left(365-l_{it}\right)}{365}=1-\frac{l_{it}}{365}. \end{array}$$

This is an upper bound on the proportion of the year that patient i is healthy. Additionally, if $d_{\text{it}}<365$ and the researcher assumes that the patient i has been hospitalized for all the unobserved $365-d_{it}$ days, then the following rate ${{h_{U}}_{it}}^{\prime }$ is an upper bound lower than ${h_{U}}_{it}:$

$$\begin{array}{r} {{h_{U}}_{it}}^{\prime }=\frac{365-l_{it}-\left({365-d}_{it}\right)}{365}=\frac{d_{it}-l_{it}}{365}. \end{array}$$

If the patient is only hospitalized for $l_{it}^{\prime }$ unobserved days where $0<l_{it}^{\prime }<\left(365-d_{it}\right)$, then ${{h_{U}}_{it}}^{\prime }$ is no longer an upper bound. In this case, ${h_{U}}_{it}$ should be used.

### 2.3 The Empirical Value of Upper Bounds in Utility Models

To illustrate the empirical value of our proposed bounds, we consider Cutler and Richardson's model (1997) that aims to measure the “health capital” of the U.S. population. Building directly on the Grossman model, they define health capital as the present discounted value of utility from healthy time over a person i’s lifetime:

$${\text{HealthCapital}}_{it}=\left(\frac{U_{H}}{U_{C}}\;\right)+E_{t}\lceil \sum_{s=0}^{\infty }\frac{H_{i,t+s}}{{\left(1+r\right)}^{s}}\rceil$$

The first term is the dollar value of a life year,^2^ and the second term is the (discounted) number of quality adjusted life years remaining at time t. The relevant quantity for us is $H_{it}$. It is the empirical counterpart of the theoretical healthy time (established in Section 2.1) and is operationalized as a quality‐adjusted life year (QALY) ranging from 0 (death) to 1 (perfect health). However, the authors assume that having 0 < $H_{it}$ < 1, that is having ill time, is possible only due to disease. Consequently, the authors assign a baseline QALY of 1 to individuals with no major chronic diseases.

Cutler and Richardson acknowledge that a measure of healthy time should include data such as “whether the person has been hospitalized for a serious illness” (1997, p. 219), but their empirical methodology for computation of QALYs does not incorporate hospitalization stays. As we demonstrated in Section 2.2, individual i’s true proportion of healthy time cannot physically exceed our proposed upper bound, ${h_{U}}_{it}=$ 1 − ($l_{it}/365$), where $l_{it}$ represents days hospitalized. If an individual without a chronic disease spends time hospitalized for an acute condition or injury ($l_{it}$ > 0), their true healthy time is strictly less than 1. By assigning a QALY of 1 in this scenario, measured $H_{it}$ exceeds the upper bound of available healthy time: ($H_{it}$ > ${h_{U}}_{it})$.

Assigning a QALY of 1 to individuals with unmeasured hospital days inflates the estimates of health capital, as seen from its definition above. Indeed, a comment by Douglas Staiger responding to the paper notes that this is an “ad hoc approach,” arguing that “there is no reason to think that QALYs are always 1 for a person without disease” (Staiger 1997, 281). Utilizing ${h_{U}}_{it}$ as an upper bound provides researchers with a corrective tool. It can help utility models provide more credible upper bounds on health capital for policy evaluation.

## 3 Clinical Medicine: Hospital Admissions

This section shifts to clinical medicine, focusing on hospital admission rates used to measure disease progression. Section 3.1 defines the clinical estimand and reviews the conventionally used hospital admission rate. Section 3.2 demonstrates that this conventional estimator may be undesirable as it inappropriately weights patients based on their follow‐up time. We propose an unweighted average rate as an alternative. Section 3.3 presents a model of hospital admissions and deaths to show the degree to which our proposed rate differs from the conventional one when patient censoring is non‐random.

### 3.1 A Conventional Hospital Admission Rate

Measuring the progression of a disease is crucial to clinical medicine. Disease progression refers to changes in the severity of the disease, particularly worsening of the disease over time. Understanding disease progression helps clinicians identify patients at higher risk of illness and tailor treatment accordingly. Hence, clinical researchers are concerned with finding quality metrics for measuring disease progression using real‐world data (Amorrortu et al. 2023). Moreover, literature on the value of healthcare has discussed the importance of measuring the severity of disease in economic assessments of healthcare resources (Lakdawalla et al. 2018; Shah 2009).

At the population level, disease progression is sometimes conflated with disease burden, as hospital admission rates are used to measure both. Disease burden refers to the impact of disease on a population or resource system (Udompap et al. 2015). Without a description of the estimand, it is hard to interpret what concept researchers aim to measure. Metcalfe et al. (2003), discussing how clinical researchers could measure hospital admissions when studying heart failure (HF), suggest using only those admissions indicative of HF progression. At the same time, they mention that readmissions informative of disease burden should also be measured.

While severe disease poses a more significant burden on the patient and healthcare system, researchers may be able to develop metrics more suitable for measuring disease progression if they are clear about the quantity they want to measure. Which type of hospital admissions to include in the measure might differ when measuring disease burden than when measuring disease progression. For instance, only hospitalizations where the disease is the primary cause may be relevant for measuring disease progression. However, all hospital admissions, regardless of the reason, contribute to the burden on the resource system for patients with the disease.

Researchers use inpatient admissions data to define various outcome measures, such as the mean number of admissions per patient or the mean number of days spent in the hospital per patient. However, a common way to define a hospital admission rate is to define it as an incidence rate, that is, counts of new admissions divided by patient‐years of follow‐up (Cummings 2019). Chen et al. (2011) studied a cohort of fee‐for‐service Medicare beneficiaries who were hospitalized for heart failure (HF). To define the admission rate, they “tabulated the total beneficiary‐months at risk (subsequently converted to beneficiary‐years) for a given year to use as the denominator, with the total number of HF hospitalizations for a given year as the numerator.” Similarly, Davy‐Mendez et al. (2019) defined admission rates “as the number of hospitalizations divided by the person‐time at risk, for the study period and each calendar year, among all patients and demographic subgroups.”

Formally, for a sample of size G this hospitalization rate is conventionally measured as

$$\begin{array}{r} H_{G}^{\prime }=\frac{365\sum_{i=1}^{G}n_{i}}{\sum_{i=1}^{G}d_{i}}, \end{array}$$

where the summations are over all patients i in the cohort, $n_{i}$ denotes the number of hospitalizations for patient i over the patient's follow‐up, and $d_{i}$ denotes the number of days of follow‐up for patient i. $\sum_{i}d_{i}$ is divided by 365 days to convert total patient‐days of follow‐up time to patient‐years of follow‐up. Our concern is that the quantity $H_{G}^{\prime }$ often lacks a clear interpretation.

Rather than use $H_{G}^{\prime }$ to measure disease progression, we suggest that clinical researchers use the revised rate ${\;H}_{G}$ that computes a simple average of individual hospital admission rates across the observed cohort of G patients:

$$\begin{array}{r} H_{G}=\frac{1}{G}\sum_{i=1}^{G}\frac{365\;n_{i}}{d_{i}} \end{array}$$

### 3.2 The Implicit Weighting Problem of the Conventional Admission Rate

We now explore the algebra associated with $H_{G}^{\prime }$ to help illuminate the strong assumptions frequently imposed and to motivate our proposed estimator $H_{G}$.

Let $D_{i},N_{i}$ be random variables—while $d_{i},n_{i}$ are realizations observable in the data. A common starting point is the *constant incidence rate* model, under which there exists a common annual *rate* λ such that

(1) $$\begin{array}{c} E\left[N_{i}|D_{i}\right]=\frac{\lambda }{365}D_{i} \end{array}$$

Under (1), $H_{G}^{\prime }$ is an unbiased estimator for λ on the realized data of follow‐up time:

$$E\left[H_{G}^{\prime }|D_{1},\text{…},D_{G}\right]=365\frac{\sum_{i=1}^{G}E\left[N_{i}|D_{i}\right]}{\sum_{i=1}^{G}D_{i}}=365\frac{\sum_{i=1}^{G}\left(\frac{\lambda }{365}D_{i}\right)}{\sum_{i=1}^{G}D_{i}}=\lambda$$

Moreover, if ${\left(N_{i},D_{i}\right)}_{i=1}^{G}$ are randomly sampled, with finite expectation and variance, and $E\left[D_{i}\right]>0$, then $H_{G}^{\prime }$ is also consistent for λ as sample size, G, grows. By the law of large numbers,

$$H_{G}^{\prime }=365\frac{G^{-1}\sum_{i}^{G}n_{i}}{G^{-1}\sum_{i}^{G}d_{i}}\to^{p}365\frac{E\left[N_{i}\right]}{E\left[D_{i}\right]}$$

Using (1),

$$365\frac{E\left[N_{i}\right]}{E\left[D_{i}\right]}=365\frac{E\left[E\left[N_{i}|D_{i}\right]\right]}{E\left[D_{i}\right]}=\lambda *365\frac{\frac{1}{365}E\left[D_{i}\right]}{E\left[D_{i}\right]}=\lambda .$$

This implies that under the constant incidence rate assumption, $H_{G}^{\prime }$ is a consistent estimator for λ:

$$H_{G}^{\prime }\to^{p}\lambda .$$

In many applications, the assumption of a common incidence rate across patients is implausible because individuals experience disease differently based on their risk factors. Cowie et al. (2002) found that among heart failure patients, older patients who were initially diagnosed as an inpatient rather than outpatient were at a higher risk of hospital admissions for worsening heart failure. A flexible specification allows for patient‐specific annual rates $\lambda_{i}$ defined as

(2) $$\begin{array}{c} E\left[N_{i}|D_{i}\right]=\frac{\lambda_{i}}{365}D_{i} \end{array}$$

Equation (3) recognizes that each patient has their own underlying risk and does not assume that a constant incidence rate (1) applies to all patients. Because the rates $\lambda_{i}$ vary across the population, we aim to measure the population average $E\left[\lambda_{i}\right]$ of these individual rates.

Consider the quantity $R_{i}≔365\frac{N_{i}}{D_{i}}$. This is the number of hospitalizations a patient has divided by their follow‐up period. See that $R_{i}$ is an unbiased estimator of that individual's incidence rate $\lambda_{i}$, because by (2)

$$E\left[R_{i}|D_{i}=d_{i}\right]=365*\frac{E\left[N_{i}|D_{i}=d_{i}\right]}{d_{i}}=\lambda_{i}.$$

The estimator $H_{G}^{\prime }$ is not a simple average of the individual patient rates $R_{i}$. Rather, $H_{G}^{\prime }$ is a weighted average of $R_{i}$ with the weights $w_{i}$ proportional to the amount of follow‐up: $w_{i}=\frac{d_{i}}{\sum_{j=1}^{G}d_{j}}$. Then,

$$H_{G}^{\prime }=365\frac{\sum_{i}^{G}n_{i}}{\sum_{i}^{G}d_{i}}=\sum_{i=1}^{G}\frac{d_{i}}{\sum_{j=1}^{G}d_{j}}r_{i}=\sum_{i}^{G}w_{i}r_{i},\text{where}\;r_{i}=365\;\frac{n_{i}}{d_{i}}.$$

These weights are positive and sum to 1, so patients with longer observed follow‐up have greater influence on the estimator, whereas patients observed only briefly have less influence. It is not apparent why clinical researchers should want to weigh individual admission rates more greatly for patients observed for a longer period in some available dataset. When studying patient measures of health such as disease progression, we expect that clinicians should want to give equal weight to all patients.^3^

Under random sampling, with finite expectation and variance, and $E\left[D_{i}\right]>0$, the law of large numbers and the previous decomposition implies:

$$\begin{array}{c} H_{G}^{\prime }=365\frac{G^{-1}\sum_{i}^{G}n_{i}}{G^{-1}\sum_{i}^{G}d_{i}}\to^{p}365\frac{E\left[N_{i}\right]}{E\left[D_{i}\right]}=\frac{E\left[\lambda_{i}D_{i}\right]}{E\left[D_{i}\right]}. \end{array}$$

When not using the *constant incidence rate* assumption (1), $H_{G}^{\prime }$ is no longer a consistent estimate of $E\left[\lambda_{i}\right]$. The difference matters especially when follow‐up time is systematically related to underlying morbidity or attrition. In such cases, the weighting scheme may skew the estimator toward the lower incidence rates of healthier patients who have longer follow‐up periods.

A sufficient condition for $H_{G}^{\prime }\to^{p}E\left[\lambda_{i}\right]$ is mean independence between the patient‐specific rate and follow‐up time, formally:

(3) $$\begin{array}{c} E\left[\lambda_{i}\right]=E\left[\lambda_{i}|D_{i}\right] \end{array}$$

Under assumption (3), $E\left[N_{i}\right]=\frac{1}{365}E\left[\lambda_{i}\right]*E\left[D_{i}\right]$ and therefore $H_{G}^{\prime }\to^{p}E\left[\lambda_{i}\right]$. Whether assumption (3) is plausible is a substantive question. In Section 3.3, we simulate scenarios in which this assumption might fail due to negative correlation between $\lambda_{i}$ and $d_{i}$.

Additionally, it is possible to test assumption (3) empirically; for example, Metcalfe et al. (2003), reporting results from Cowie et al. (2002), show that follow‐up and incidence rates are not independent, and are unlikely to be mean independent. That is, $H_{G}^{\prime }{↛}^{p}E\left[\lambda_{i}\right]$. Our primary motivation for proposing the estimator $H_{G}$ is that it is consistent for $E\left[\lambda_{i}\right]:$

$$\begin{array}{r} H_{G}=\frac{1}{G}\sum_{i=1}^{G}R_{i}=\frac{1}{G}\sum_{i=1}^{G}\frac{365\;n_{i}}{d_{i}}\to^{p}E\left[\lambda_{i}\right] \end{array}$$

Unlike the conventional rate $H_{G}^{\prime }$, $H_{G}$ guarantees this consistency regardless of whether follow‐up time and individual incidence rates are mean independent (assumption 3). Therefore, it is a better alternative when patients have heterogeneous incidence rates.

Further, the rate $H_{G}$ gives equal weight to each patient in the period that the patient is observed, rather than giving more weight to patients who are observed for longer periods. It demonstrates that carefully specifying estimands helps researchers formulate estimators with properties that align well with the quantity of interest. The incoherent weighting scheme of the common rate ${H_{G}}^{\prime }$ cannot be justified by the goal of measuring disease progression at the patient‐level. $H_{G}$ is a better alternative because it measures the average disease severity in the population, which is a closer proxy for disease progression.

### 3.3 $H_{G}^{\prime }$ Versus $H_{G}$: Modeling the Relationship Between Hospital Admissions and Death

In cases where patient‐time $d_{i}$ is collected from administrative datasets with potentially non‐random censoring (such as insurance claims data), the independence between follow‐up time and hospital admission rates in Equation (3) might fail. For example, a patient experiencing many adverse health events might switch to a more comprehensive health plan. It might lead to a relationship where if $\lambda_{i}$ is large (indicating a frequently hospitalized individual) then $D_{i}$ might systematically be smaller. Further, in situations such as death due to disease, patients may leave the dataset, again creating negative dependence between $\lambda_{i}$ and $D_{i}.$ We model and analyze this scenario in detail.

To study how the relationship between death and hospitalizations affects ${H_{G}}^{\prime }$ and $H_{G}$, we analyze a model representing 10‐year of hospital admissions data for a “typical” Medicare patient. That is, patients experience a hospitalization rate of $\lambda_{i}∼\text{LogNormal}\left(\log\left(0.9\frac{\text{hosps}}{\text{year}}\right)-\frac{\tau^{2}}{2},\tau^{2}\right)$. This matches the expected rate of 0.9 hospitalizations/patient year found in Medicare data (Walsh et al. 2010). The lognormal distribution was chosen so that the rate is always positive. τ is set to 0.5 for concreteness. Correspondingly, 95% of hospitalization rates fall between 0.30 and 2.12 hospitalizations a year.^4^

For recording the risk of patient death from a hospitalization, we use a 30‐day post‐hospitalization mortality rate for Medicare patients: 8.17% (Centers for Medicare and Medicaid Services (CMS) 2023). We assume this rate is constant across patients. Nevertheless, patients who are sicker and thus have more hospital admissions will die and leave the dataset sooner, creating a negative relationship between $\lambda_{i}$ and $d_{i}$. Patients who are observed longer tend to be healthier patients.

To study the situation, we define the 30‐day post‐hospitalization mortality rate as a baseline of 8.17% multiplied by a scaling factor, γ. This scaling factor allows us to adjust the baseline mortality rate to test difference scenarios. We evaluate the model across five γ values: 0,0.5,1,2,4. We assume that γ is constant across patients overtime and takes on nonnegative values, although it could be allowed to vary across patients, overtime, or take on negative values. We present our modeling results in Table 1. Overall, the analytical results show that as γ increases, there is more negative correlation between $\lambda_{i}$ and $D_{i}$. When γ=0, there is zero correlation: $\text{Corr}\left(\lambda_{i},d_{i}\right)=0$. At our most realistic scenario, γ=1, the correlation becomes −0.30. For γ=4, the correlation grows in magnitude to −0.37. In the case of γ=0, which satisfies assumption (3), both $H_{G}^{\prime }$ and $H_{G}$ converge to the same rate of 0.9. For all other γ, assumption (3) no longer holds, and while $H_{G}$ remains at 0.9 as expected, the traditional hospitalization rate $H_{G}^{\prime }\to^{p}0.75$. Further details are described in Supporting Information S1: Appendix A.

**TABLE 1:** Comparison of traditional and proposed hospital admission rate estimators.

	γ=0	γ=0.5	γ=1	γ=2	γ=4
$H^{\prime }\to^{p}\frac{E\left[\lambda_{i}D_{i}\right]}{E\left[D_{i}\right]}$	0.9	0.86	0.83	0.79	0.75
$H\to^{p}E\left[\lambda_{i}\right]$	0.9	0.9	0.9	0.9	0.9
$Corr\left(\lambda_{i},D_{i}\right)$	0	−0.24	−0.30	−0.35	−0.37
Satisfies Equation (3)	Yes	No	No	No	No

*Note:* This table compares the probability limits of the traditional hospital admission rate estimator ($H^{\prime })$ against the proposed alternative (H), under varying 30‐day post‐hospitalization mortality rates. The parameter γ scales the baseline mortality rate of 8.17%. Here, γ=0 represents no relationship between death and hospitalization, whereas γ=1 represents the population average. The table also presents the correlation between the underlying incidence rate, $\lambda_{i},$ and total follow‐up time, $D_{i}$. The last row indicates whether the Equation 3 (mean independence between $\lambda_{i}$ and $D_{i}$) is satisfied for each γ value. Further details are in Supporting Information S1: Appendix A.

Our choice to look 10‐year into the future is not innocuous. While the choice of 10‐year may or may not be representative of a cohort study, it affects the correlation observed. While $\lambda_{i}$ is constant in all time periods—in our model the correlation $\text{corr}\left(\lambda_{i},D_{i}\right)$ will grow in magnitude as simulated time increases. In our baseline setting, with γ=1, a quadratic approximation given a function of total follow up time T is $\text{corr}\left(\lambda_{i},D_{i}\left(T\right)\right)\approx -0.05T+0.002T^{2}$. The exact formula is given in Supporting Information S1: Appendix A. Notably, follow‐up time affects the correlation between $\lambda_{i}$ and $D_{i}$, since non‐random censoring has more ability to influence and undermine incidence rates as the study duration increases. In our example, the endogeneity of $\lambda_{i}$ and $d_{i}$ occurs through the internal mechanism of hospital deaths. This same effect could be caused by unobserved covariates such as frailty, affecting both the rate of hospitalizations and the length of follow up. Overall, in this model, we have shown that due to the endogeneity of $\lambda_{i}$ and $d_{i}$, assumption (3) is violated. Therefore, we recommend using the estimator $H_{G}$ over ${H_{G}}^{\prime }$. It is equally simple but remains consistent for $E\left[\lambda_{i}\right]$ even when patients are believed to have varying incidence rates.

## 4 Clinical Medicine: Hospital Readmissions

We examine risk‐standardized readmission rates as a measure of hospital performance and quality. Section 4.1 introduces the CMS Hospital‐Wide All‐Cause Readmission (HWR) measure. Section 4.2 mathematically demonstrates that the CMS's standardized readmission ratio can unreasonably penalize hospitals for treating a sicker case mix. To correct this, Section 4.3 draws on econometric production theory to propose ranking hospitals based on their intercepts in the CMS risk‐standardization model, which accurately isolate unobserved efficiency.

### 4.1 The Hospital‐Wide All‐Cause Readmission (HWR) Rate: Background

Researchers and administrative agencies concerned with the quality of care in inpatient healthcare facilities attempt to measure hospital performance to assess and compare hospitals. Comparison between hospitals is complicated by differences in the patient case mix (age and illness severity characteristics) and hospital service mix (procedures and services offered by hospitals) (Horwitz et al. 2012).

To compare performance across hospitals serving Medicare patients and adjust for differences in case mix, the US Centers for Medicare & Medicaid Services (CMS) relies on risk‐standardized measures. As part of its Hospital Readmissions Reduction Program (HRRP), it publicly reports its Hospital‐Wide All‐Cause Readmission (HWR) measure for eligible acute care hospitals because it is mandated to “reduce the payments” for “excess readmissions” (U.S. Congress 2010). The HWR measure is a hospital‐level 30‐day risk‐standardized readmission rate based on unplanned all‐cause readmissions. We provide a broader discussion of the clinical validity and financial incentives associated with the HRRP in Supporting Information S1: Appendix B.

### 4.2 Calculation of the Hospital‐Wide All‐Cause Readmission (HWR) Rate

CMS calculates the HWR by estimating a hierarchical logistic regression model. This is done as follows. Let $Y_{ij}$ represent a binary outcome for whether a patient i is readmitted for an unplanned readmission at hospital j within 30 days. Let $Z_{ij}$ represent a vector of patient‐specific covariates or risk factors. Let $\alpha_{j}$ be the hospital‐specific intercept, assumed to follow a cross‐hospital normal distribution with mean μ and variance $\tau^{2}$. Then, $Y_{ij}$ is assumed to be related to the covariates as follows:

$$\begin{array}{rr} & \ln\left(\frac{\mathrm{Pr}\left(Y_{ij}=1|Z_{ij},\omega_{j}\right)}{1-\mathrm{Pr}\left(Y_{ij}=1|Z_{ij},\omega_{j}\right)}\right)=\alpha_{j}+\beta Z_{ij},\\ \text{where}\;\alpha_{j} & =\mu +\omega_{j}\;\text{and}\;\omega_{j}∼N\left(0,\tau^{2}\right). \end{array}$$

After using this hierarchical logistic regression model to obtain parameter estimates, the ratio of predicted readmissions to the number of expected readmissions is calculated as

$$\begin{array}{r} \hat{s_{j}}=\frac{\sum_{i=1}^{n_{j}}f\left(\hat{\alpha_{j}}+\hat{\beta }Z_{ij}\right)\;}{\sum_{i=1}^{n_{j}}f\left(\hat{\mu }+\hat{\beta }Z_{ij}\right)}, \end{array}$$

where $f\left(x\right)=\frac{1}{1+e^{-x}}$ is the standard logistic function , and $n_{j}$ denotes the number of admissions at hospital j. This is the “standardized readmission ratio” for hospital j. It is computed to compare the “observed” performance of a particular hospital to the “expected” performance of an average hospital (DeBuhr et al. 2024). It is multiplied by the national crude readmission rate to yield a risk‐standardized readmission rate, which is the HWR measure. For example, a hospital with a 10% readmission rate that is expected to have a readmission rate of 20% is given a standardized ratio of ${\hat{s}}_{j}=0.5.$ Given that the national crude readmission rate is approximately 14%, CMS would report that the hospital has a 7% standardized readmission rate.

Under this model, the hospital‐specific intercepts $\alpha_{j}$ capture differences in readmissions across hospitals associated with unobservable hospital characteristics. The hospital‐specific intercept is estimated while conditioning on patient covariates and reflects differences in hospital quality that are independent of the observable case mix.

Therefore, we argue that CMS should rank hospitals based on their intercepts $\alpha_{j}$ rather than the standardized readmission ratio described above. Hospital rankings based on intercepts may differ from those based on standardized readmission ratios. In Supporting Information S1: Appendix C, we formally show that when comparing the performance of any two hospitals indexed as 1 and 2, drawn from a broader sample, each with one patient and where μ > $\hat{\;\alpha_{1}}$ > $\hat{\alpha_{2}}$, making the patient worse off in Hospital 2 can produce a hospital ranking that is inconsistent with the ranking based on intercepts $\hat{\alpha }$ (Proposition C1). Specifically, Hospital 1 can end up ranking better in performance (lower $\hat{s})$ than Hospital 2 when using $\hat{s},$ despite ranking worse when using $\hat{\alpha }$ (higher $\hat{\alpha })$.

Since CMS determines payment reductions by comparing a hospital's standardized readmission ratio to the median ratio of peer group hospitals, Table 2 illustrates that a worse patient case‐mix at Hospital 2 could lead to a greater payment reduction due to an increased standardized readmission ratio. Thus, hospitals that perform better than average in preventing readmissions may be penalized for a poor case mix when the standardized readmission ratio is used to assess performance.

**TABLE 2:** Standardized readmission ratios for high‐quality hospitals across patient risk factors.

Hospital $\hat{\alpha }$	$\hat{s}$ when $\hat{\beta }Z=1$	$\hat{s}$ when $\hat{\beta }Z=2.5$
${\hat{\alpha }}_{1}=0.9$	0.988	0.997
${\hat{\alpha }}_{2}=0.7$	0.960	0.990

*Note:* This table compares the standardized readmission ratio of two hospitals serving a patient when the patient has different risk factors. Both hospitals have $\hat{\alpha }$ values below μ, the average quality, which equals 1. Hospital 1 has a higher $\hat{\alpha }$ and is therefore of lower quality than Hospital 2. Ratio values on the diagonal indicate that an increase in $\hat{\beta }Z$ for the patient in Hospital 2 results in a ratio that exceeds that of Hospital 1 for the original $\hat{\beta }Z$.

We prove an analogous result that a hospital performing worse than average in preventing readmissions can, conversely, benefit from a poor case‐mix (Supporting Information S1: Appendix C, Proposition C2). In Table 3, we show that when comparing two hospitals where $\hat{\;\alpha_{1}}>\hat{\;\alpha_{2}}>\;\mu$, Hospital 1—despite having lower quality (higher $\hat{\alpha })-$ ranks better than Hospital 2 (lower $\hat{s})$ when the patient case‐mix at Hospital 1 worsens (i.e., an increase in $\hat{\beta }Z_{11}$) and performance is assessed using the standardized readmission ratio.

**TABLE 3:** Standardized readmission ratios for low‐quality hospitals across patient risk factors.

Hospital $\hat{\alpha }$	$\hat{s}$ When $\hat{\beta }Z=1$	$\hat{s}$ When $\hat{\beta }Z=3$
${\hat{\alpha }}_{1}=1.5$	1.049	1.007
${\hat{\alpha }}_{2}=1.1$	1.011	1.002

*Note:* This table compares the standardized readmission ratio of two hospitals serving a patient when the patient has different risk factors. Both hospitals have $\hat{\alpha }$ values above μ, the average quality, which equals 1. Hospital 1 has a higher $\hat{\alpha }$ and is therefore of lower quality than Hospital 2. Ratio values on the diagonal indicate that an increase in $\hat{\beta }Z$ for the patient in Hospital 1 results in a ratio lower than that of Hospital 2 for the original $\hat{\beta }Z$.

Thus, by using hospital‐specific intercepts to rank hospitals, CMS would evaluate them based on their unobserved efficiency in preventing readmissions—the quantity of interest.

### 4.3 Hospital Intercepts as Unobserved Efficiency in Risk‐Standardized Readmission Rates

Treating the unit‐specific intercept as a parameter of interest, rather than as a nuisance parameter, aligns with a long‐standing tradition in applied econometrics (Chamberlain 1984; Hoch 1962; Mundlak 1961). Yet, in evaluating hospital performance, where the primary objective is to isolate a hospital's contribution to readmission risk from its patient case mix, our proposal to use hospital‐specific intercepts to rank quality is novel. The biostatistical literature on provider profiling (comparing the quality of care among providers) employs hierarchical logistic regression models (Daniels and Gatsonis 1999) and recognizes that a “variable intercept indicates interhospital differences in baseline … rates” (Normand et al. 1997). But it falls short of using the intercept itself as the primary metric for ranking hospitals.

This approach to the intercept, treating it not as a shock but as a fixed characteristic, has roots in production theory. In that literature, Mundlak (1961) and Hoch (1962) explicitly isolated the unit‐specific intercept to measure unobserved “management” and “technical efficiency” of firms, treating it as a parameter of interest in the estimation of production functions. Building on this, Chamberlain (1984) later formalized the unit fixed effect as latent quality that the producer bases decisions upon, even though it remains unobserved to the researcher.

When modeling readmissions, the hospital‐specific intercepts, $\alpha_{j},$ act identically to this firm‐specific constant. Hospital intercepts capture the latent institutional quality that shifts the readmission probability conditional on the patient's observable state. Therefore, within the context of the hierarchical logistic regression model currently employed by the CMS, ranking these intercepts provides a theoretically sound estimator of the parameter CMS seeks to regulate: the hospital's intrinsic efficiency at reducing unplanned readmissions.

## 5 Conclusion

Across health economics and clinical medicine, researchers frequently use hospitalization data to estimate concepts such as healthy time, disease progression, and hospital performance. As our three applications demonstrate, heuristically relying on conventional rate estimators can lead to upward biases in welfare estimations, inappropriate patient weighting in incidence rates, or unreasonable institutional penalties. By defining the estimand of interest first, researchers can design estimators that measure what they intend to measure.

Conceptual clarity is important because cross‐disciplinary borrowing can lead to ambiguous definitions. The same hospitalization rate is sometimes used to measure different quantities in different fields. Readmission rates measure disease progression and hospital performance in clinical research. Rates may be defined differently but still be referred to by the same name. Readmission rates can refer to the ratio of readmissions to index hospital admissions, but they can also refer to the ratio of predicted readmissions to expected readmissions when assessing hospital performance.

The heuristic use of existing rate definitions in health research underscores our message: researchers should elaborate on the definitions and motivations behind their estimators to help clarify them for readers. Relatedly, the appraisal of estimators should give central importance to the quantity that the researchers aim to measure. Researchers may realize that there are better measures or more than one estimator that they could use to measure the quantity of interest.

## Funding

The authors have nothing to report.

## Conflicts of Interest

The authors declare no conflicts of interest.

## Supporting information


Supporting Information S1

Supporting Information S2

Supporting Information S3

## Data Availability

The data that supports the findings of this study are available in the supplementary material of this article.

## Endnotes

^1^
We depart from Grossman's notation, in which time devoted to work is represented as TW.

^2^
The ratio of marginal utilities, UHUC, is the marginal rate of substitution between healthy time and other consumption goods. Cutler and Richardson (1997) interpret it as “the dollar value of an additional life year in perfect health.”

^3^
Note that the implicit weighting scheme used in $H_{G}^{\prime }$ contradicts the intuition behind inverse probability weighting, where patients with shorter observation periods receive higher weights, representing the inverse of the weights used in $H_{G}^{\prime }:$ $1/(\frac{d_{i}}{\sum_{j=1}^{G}d_{j}})$. In other words, the implicit weighting scheme of the traditional hospital rate is the exact inverse of the weights used in inverse probability weighting to handle loss to follow‐up (Mansournia and Altman 2016). Because clinical researchers seek to understand typical disease progression by giving equal weight to every patient, inverse weighting is largely irrelevant in estimating inpatient admission rates as it distorts the average by upweighting those with shorter observation periods.

^4^
To see this, note that $P\left(0.30\leq \lambda_{i}\leq 2.12\right)=\Phi \left(\frac{\log\left(2.12\right)-\left(\log\left(0.9\right)-\frac{0.5^{2}}{2}\right)}{0.5}\right)-\Phi \left(\frac{\log\left(0.30\right)-\left(\log\left(0.9\right)-\frac{0.5^{2}}{2}\right)}{0.5}\right)\;\approx \;\Phi \left(1.96\right)-\Phi \left(-1.96\right)=95\%$.
