# Supplementary material for: Assessing the Estimands and Estimates of Hospitalization Rates in Health Economics and Clinical Medicine
Source: Health Econ. 2026 Jun 18;35(9):1397–405. doi: 10.1002/hec.70117 (PMC13428625; doi:10.1002/hec.70117)
Supplement: Supplementary file 1 — Supporting Information S1 [file HEC-35-1397-s003.docx]

# Appendix A

In this appendix, we formalize the model from Section 3.3 and present the closed-form expressions used. The setup is described first.

For simplicity we use a discrete setup (instead of continuous) where we model events at the patient-month level. In the absence of death, the total follow-up time for each patient is $T=$120-months, equivalent to 10-years. We index patients by subscript $i$, and the month since cohort inclusion by $t\in\left\{ 1,\ldots,120 \right\}$. We denote the number of events in each patient month as $n_{it}\in\mathbb{Z}^{+}$, and a 0/1 indicator if a patient is enrolled in each patient month as $d_{it}\in\left\{ 0,1 \right\}$. As in the main text, we denote total hospitalizations and total follow up as $N_{i}=\sum_{t=1}^{120} n_{it}$ and $D_{i}=\sum_{t=1}^{120} d_{it}$. In the main text, results are given in terms of patient-year, in the appendix we use both patient-month and patient-year, making clear which is used in each instance.

We now describe the number of hospitalizations, $n_{it}$. Overall, we center our hospitalization rate around 0.9 hospitalizations per patient-year, that is 0.075 per patient-month. This number was chosen to match the rate for the year 2005 in a Medicare population (Walsh et al., 2010) .^[[1]](#footnote-1)^ The number of hospitalizations, $n_{it}$, must logically be an integer. Certain data generating processes like Poisson satisfy this mechanically. To achieve a more general setup we choose a continuous form modeling the expected number of hospitalizations as $x\mathbb{\in R}$. Then, we map that choice onto $\mathbb{Z}^{+}$ through the floor function, i.e., $n_{it}= \left\lfloor x \right\rfloor$. We elaborate more about this floor function shortly. To make the bias introduced by a floor function arbitrarily small we introduce a *nuisance* scale factor c. We now provide some analytical details:

- $c$ is an arbitrarily large number,
- $\mathbb{P(}n_{it}=0|d_{it}=0)=1$,
- If $d_{it}=1$, then $n_{it}=\left\lfloor c\cdot\lambda_{i} \right\rfloor,$ and
- $\lambda_{i}\sim Lognormal\left( \log\left( 0.075 \right)-\frac{{0.5}^{2}}{2},{0.5}^{2} \right)$.

The choice of $\tau=0.5$ for the distribution of $\lambda_{i}$ is described in the main text. It is unimportant and was chosen arbitrarily. For simplicity we don’t include any time-varying multiplicative error term, $e_{it}$, but this could easily be incorporated if the multiplicative error is independent and identically distributed (i.i.d.) across both $i$ and $t$, with a mean of 1.

The floor function, while important for ensuring that our variable, $n_{it}$, is an integer, creates difficulties (see, for example, that $\mathcal{R}\left( E[\lambda_{i}] \right)=\left\lfloor0.075 \right\rfloor=0$). To overcome these difficulties, we apply the “trick” of multiplying by an arbitrarily large constant, then dividing by the same constant later. As c increases the approximation improves, i.e, $\forall x\mathbb{\in R,}\lim_{c\to\infty} \frac{1}{c}\left\lfloor c\cdot x \right\rfloor=x$. (We use the shorthand $\to^{c}$ for $\lim_{c\to\infty}$to reduce notation). In our final table we adjust our counts of $N_{i}$ and $\lambda_{i}$ into units of 1 patient-year by multiplying by 12 and dividing by $c$.

The following describes the “censoring” mechanism of death and how it impacts follow up. Let $\gamma\cdot8.17\%$ denote the 30-day post-hospitalization mortality rate, where $\gamma\in\{0,0.5,1,2,4\}$ is as described in the main text and 8.17% is sourced from (Centers for Medicare & Medicaid Services [CMS], 2023).^[[2]](#footnote-2)^ Note that,

- $d_{i,1}=1$
- $\mathbb{P(}d_{i,t+1}=1|d_{i,t}=0)=0$
- $\mathbb{P(}d_{i,t+1}=1|d_{it}=1,n_{it}=0)=1$
- $\mathbb{P(}d_{i,t+1}=1|d_{it}=1,n_{it}\geq1)=\left( 1-\frac{1}{c}\cdot\gamma\cdot0.0817 \right)^{n_{it}}\to^{c}e^{-\gamma\cdot0.0817\cdot\lambda_{i}}$

We now describe the analytical results shown in Table 1:

**Step 1:** $\boldsymbol{E[}\boldsymbol{D}_{\boldsymbol{i}}\boldsymbol{]}$

$$E\left[ D_{i} | \lambda_{i}=\lambda\right]=\sum_{t=1}^{120} E\left[ d_{it} | \lambda_{i}=\lambda\right]=\sum_{t=2}^{120} \mathbb{P[}d_{it}=1|d_{i\left\{ t-1 \right\}}=1,\lambda_{i}=\lambda]$$

$$=\sum_{t=1}^{120} \left( 1-\frac{1}{c}\cdot\gamma\cdot0.0817 \right)^{\left\lfloor c\cdot\lambda\right\rfloor\cdot(t-1)}=\frac{1-\left( 1-\frac{1}{c}\cdot\gamma\cdot0.0817 \right)^{120\cdot\left\lfloor c\cdot\lambda\right\rfloor}}{{1-\left( 1-\frac{1}{c}\cdot\gamma\cdot0.0817 \right)}^{\left\lfloor c\cdot\lambda\right\rfloor}}$$

Integrating over the lognormal distribution of $\lambda$ yields,

$$E\left[ D_{i} \right]=\int_{0}^{\infty} \frac{1-\left( 1-\frac{1}{c}\cdot\gamma\cdot0.0817 \right)^{120\cdot\left\lfloor c\cdot\lambda\right\rfloor}}{{1-\left( 1-\frac{1}{c}\cdot\gamma\cdot0.0817 \right)}^{\left\lfloor c\cdot\lambda\right\rfloor}}f_{\lambda}\left( \lambda\right)d\lambda\to^{c}\int_{0}^{\infty} \frac{1-e^{-120\cdot\gamma\cdot0.0817\cdot\lambda}}{1-e^{-\gamma\cdot0.0817\cdot\lambda}}f_{\lambda}\left( \lambda\right)d\lambda$$

**Step 2:** $\boldsymbol{E}\left[ \frac{\boldsymbol{N}_{\boldsymbol{i}}}{\boldsymbol{c}} \right]$

Since,

$N_{i}=\sum_{t=1}^{120} n_{it}=\left\lfloor c\cdot\lambda\right\rfloor D_{i}$,

we have,

$E\left[ N_{i} | \lambda_{i}=\lambda\right]=\left\lfloor c\cdot\lambda\right\rfloor E[D_{i}|\lambda_{i}=\lambda]$.

Therefore,

$E\left[ N_{i} | \lambda_{i}=\lambda\right]= \left\lfloor c\cdot\lambda\right\rfloor\frac{1-\left( 1-\frac{1}{c}\cdot\gamma\cdot0.0817 \right)^{120\cdot\left\lfloor c\cdot\lambda\right\rfloor}}{{1-\left( 1-\frac{1}{c}\cdot\gamma\cdot0.0817 \right)}^{\left\lfloor c\cdot\lambda\right\rfloor}}$,

and

$E\left[ \frac{N_{i}}{c} | \lambda_{i}=\lambda\right]=\frac{\left\lfloor c\cdot\lambda\right\rfloor}{c}\frac{1-\left( 1-\frac{1}{c}\cdot\gamma\cdot0.0817 \right)^{120\cdot\left\lfloor c\cdot\lambda\right\rfloor}}{{1-\left( 1-\frac{1}{c}\cdot\gamma\cdot0.0817 \right)}^{\left\lfloor c\cdot\lambda\right\rfloor}}$.

Integrating over the lognormal distribution of $\lambda$ yields,

$$E\left[ \frac{N_{i}}{c} \right]=\int_{0}^{\infty} \frac{\left\lfloor c\cdot\lambda\right\rfloor}{c}\frac{1-\left( 1-\frac{1}{c}\cdot\gamma\cdot0.0817 \right)^{120\cdot\left\lfloor c\cdot\lambda\right\rfloor}}{{1-\left( 1-\frac{1}{c}\cdot\gamma\cdot0.0817 \right)}^{\left\lfloor c\cdot\lambda\right\rfloor}}f_{\lambda}\left( \lambda\right)d\lambda\to^{c}\int_{0}^{\infty} \lambda\frac{1-e^{-120\cdot\gamma\cdot0.0817\cdot\lambda}}{1-e^{-\gamma\cdot0.0817\cdot\lambda}}f_{\lambda}\left( \lambda\right)d\lambda.$$

Above, we used the principal benefit of introducing the scaling factor c, as $\frac{\left\lfloor c\cdot\lambda\right\rfloor}{c}\to^{c}\lambda$. Without this scale factor, analysis would change substantially in ways that are uninteresting to the content of Section 3.3.

**Step** **3:** $\boldsymbol{E}\left[ \frac{\boldsymbol{N}_{\boldsymbol{i}}}{\boldsymbol{c}} \right]\boldsymbol{\to}^{\boldsymbol{c}}\boldsymbol{E[}\boldsymbol{\lambda}_{\boldsymbol{i}}\boldsymbol{D}_{\boldsymbol{i}}\boldsymbol{]}$

This is clear, but for completeness note,

$$E\left[ \lambda_{i}D_{i} \right]=\int_{0}^{\infty} \lambda\cdot E\left[ \lambda_{i}D_{i} | \lambda_{i}=\lambda\right]f_{\lambda}\left( \lambda\right)d\lambda=\int_{0}^{\infty} \lambda\frac{1-\left( 1-\frac{1}{c}\cdot\gamma\cdot0.0817 \right)^{120\cdot\left\lfloor c\cdot\lambda\right\rfloor}}{{1-\left( 1-\frac{1}{c}\cdot\gamma\cdot0.0817 \right)}^{\left\lfloor c\cdot\lambda\right\rfloor}}f_{\lambda}\left( \lambda\right)d\lambda$$

$\to^{c}\int_{0}^{\infty} \lambda\frac{1-e^{-120\cdot\gamma\cdot0.0817\cdot\lambda}}{1-e^{-\gamma\cdot0.0817\cdot\lambda}}f_{\lambda}\left( \lambda\right)d\lambda=E\left[ \frac{N_{i}}{c} \right]$.

**Step 4:** $\boldsymbol{Cov(}\boldsymbol{\lambda}_{\boldsymbol{i}}\boldsymbol{,}\boldsymbol{D}_{\boldsymbol{i}}\boldsymbol{)}$

Since

$$Cov\left( \lambda_{i},D_{i} \right)=E\left[ \lambda_{i}D_{i} \right]-E\left[ \lambda_{i} \right]E[D_{i}]$$

we have that,

$$Cov\left( \lambda_{i},D_{i} \right)=\int_{0}^{\infty} \lambda\frac{1-e^{-120\cdot\gamma\cdot0.0817\cdot\lambda}}{1-e^{-\gamma\cdot0.0817\cdot\lambda}}f_{\lambda}\left( \lambda\right)d\lambda-0.075\cdot\int_{0}^{\infty} \frac{1-e^{-120\cdot\gamma\cdot0.0817\cdot\lambda}}{1-e^{-\gamma\cdot0.0817\cdot\lambda}}f_{\lambda}\left( \lambda\right)d\lambda$$

**Step 5:** $\boldsymbol{Var}\left( \boldsymbol{\lambda}_{\boldsymbol{i}} \right)$ **and** $\boldsymbol{Var(}\boldsymbol{D}_{\boldsymbol{i}}\boldsymbol{)}$

$$Var\left( \lambda_{i} \right)={0.075}^{2}\left( e^{{0.5}^{2}}-1 \right)\approx0.0016$$

Now for $Var\left( D_{i} \right)$, note the equivalence,

$Var\left( D_{i} \right)=E\left[ D_{i}^{2} \right]-E\left[ D_{i} \right]^{2}$.

And,

$D_{i}^{2}=\sum_{t=1}^{120} \sum_{u=1}^{120} \mathbb{I[}D_{i}\geq t,D_{i}\geq u]$,

so $E\left[ D_{i}^{2} | \lambda_{i}=\lambda\right]=\sum_{t=1}^{120} \sum_{u=1}^{120} \left( 1-\frac{1}{c}\cdot\gamma\cdot0.0817 \right)^{\left\lfloor c\cdot\lambda\right\rfloor\cdot(\max\left( t,u \right)-1)}$

$$=\sum_{k=1}^{120} (2k-1)\left( 1-\frac{1}{c}\cdot\gamma\cdot0.0817 \right)^{\left\lfloor c\cdot\lambda\right\rfloor\cdot(k-1)}.$$

Therefore,

$$E\left[ D_{i}^{2} \right]=\int_{0}^{\infty} \left[ \sum_{k=1}^{120} \left( 2k-1 \right)\left( 1-\frac{1}{c}\cdot\gamma\cdot0.0817 \right)^{\left\lfloor c\cdot\lambda\right\rfloor\cdot\left( k-1 \right)} \right]f_{\lambda}\left( \lambda\right)d\lambda.$$

We arrive at,

$$Var\left( D_{i} \right)\to^{c}\int_{0}^{\infty} \left[ \sum_{k=1}^{120} \left( 2k-1 \right)e^{-\left( k-1 \right)\gamma\cdot0.0817\cdot\lambda} \right]f_{\lambda}\left( \lambda\right)d\lambda-\left( \int_{0}^{\infty} \frac{1-e^{-120\cdot\gamma\cdot0.0817\cdot\lambda}}{1-e^{-\gamma\cdot0.0817\cdot\lambda}}f_{\lambda}\left( \lambda\right)d\lambda\right)^{2}.$$

**Step 6:** $\boldsymbol{Corr(}\boldsymbol{\lambda}_{\boldsymbol{i}}\boldsymbol{,}\boldsymbol{D}_{\boldsymbol{i}}\boldsymbol{)}$**, and correlation using maximum follow-up time T,** $\boldsymbol{Corr(}\boldsymbol{\lambda}_{\boldsymbol{i}}\boldsymbol{,}\boldsymbol{D}_{\boldsymbol{i}}\boldsymbol{(T))}$**,**

Using $Corr\left( \lambda_{i},D_{i} \right)=\frac{Cov(\lambda_{i},D_{i})}{\sqrt{Var\left( \lambda_{i} \right)Var(D_{i})}}$, we arrive at a closed form solution

$$Corr\left( \lambda_{i},D_{i} \right)=\frac{\int_{0}^{\infty} \lambda\frac{1-e^{-120\cdot\gamma\cdot0.0817\cdot\lambda}}{1-e^{-\gamma\cdot0.0817\cdot\lambda}}f_{\lambda}\left( \lambda\right)d\lambda-0.075\cdot\int_{0}^{\infty} \frac{1-e^{-120\cdot\gamma\cdot0.0817\cdot\lambda}}{1-e^{-\gamma\cdot0.0817\cdot\lambda}}f_{\lambda}\left( \lambda\right)d\lambda}{\sqrt{\left( {0.075}^{2}\left( e^{{0.5}^{2}}-1 \right) \right)\cdot\left( \int_{0}^{\infty} \left[ \sum_{k=1}^{120} \left( 2k-1 \right)e^{-\left( k-1 \right)\gamma\cdot0.0817\cdot\lambda} \right]f_{\lambda}\left( \lambda\right)d\lambda-\left( \int_{0}^{\infty} \frac{1-e^{-120\cdot\gamma\cdot0.0817\cdot\lambda}}{1-e^{-\gamma\cdot0.0817\cdot\lambda}}f_{\lambda}\left( \lambda\right)d\lambda\right)^{2} \right)}}$$

There is nothing unique about $T=120$ here. Thus, for any maximum follow-up T, we arrive at,

$$Corr\left( \lambda_{i},D_{i}(T) \right)=\frac{\int_{0}^{\infty} \lambda\frac{1-e^{-T\cdot\gamma\cdot0.0817\cdot\lambda}}{1-e^{-\gamma\cdot0.0817\cdot\lambda}}f_{\lambda}\left( \lambda\right)d\lambda-0.075\cdot\int_{0}^{\infty} \frac{1-e^{-T\cdot\gamma\cdot0.0817\cdot\lambda}}{1-e^{-\gamma\cdot0.0817\cdot\lambda}}f_{\lambda}\left( \lambda\right)d\lambda}{\sqrt{\left( {0.075}^{2}\left( e^{{0.5}^{2}}-1 \right) \right)\cdot\left( \int_{0}^{\infty} \left[ \sum_{k=1}^{T} \left( 2k-1 \right)e^{-\left( k-1 \right)\gamma\cdot0.0817\cdot\lambda} \right]f_{\lambda}\left( \lambda\right)d\lambda-\left( \int_{0}^{\infty} \frac{1-e^{-T\cdot\gamma\cdot0.0817\cdot\lambda}}{1-e^{-\gamma\cdot0.0817\cdot\lambda}}f_{\lambda}\left( \lambda\right)d\lambda\right)^{2} \right)}} .$$

In figure A1, we graph the correlation as a function of $\gamma$ and T. It is worth noting that the relationship between the correlation and $T$ is not necessarily monotonic. As seen in figure A1, for large enough T and $\gamma=4$, the magnitude of correlation decreases as T increases. Also, note that the quadratic approximation presented in the main text for $\gamma=1$ is for illustration purposes. It is arrived at by least squares of a quadratic function with a zero intercept$.$

**Step 7:** $\boldsymbol{E}\left[ \boldsymbol{H}_{\boldsymbol{G}} \right]$ **and** $\boldsymbol{E[}\boldsymbol{H}_{\boldsymbol{G}}^{\boldsymbol{'}}\boldsymbol{]}$

This is simple recalling from the main text and the lognormal distribution of $\lambda_{i}$

- $E\left[ H_{G} \right]=E\left[ \lambda_{i} \right]=0.075$,
  - See that $E\left[ \frac{1}{G}\sum_{i}^{G} R_{i} \right]=E\left[ R_{i} \right]=E\left[ \frac{N_{i}}{c\cdot D_{i}} \right]=\int_{0}^{\infty} E\left[ \frac{N_{i}}{c\cdot D_{i}}|\lambda_{i}=\lambda\right]f_{\lambda}\left( \lambda\right)d\lambda=\int_{0}^{\infty} \frac{\left\lfloor c\cdot\lambda\right\rfloor}{c}E\left[ \frac{D_{i}}{D_{i}}|\lambda_{i}=\lambda\right]f_{\lambda}\left( \lambda\right)d\lambda$, as $N_{i}=\left\lfloor c\cdot\lambda_{i} \right\rfloor D_{i}$

$$\to^{c}\int_{0}^{\infty} \lambda f_{\lambda}\left( \lambda\right)d\lambda=E\left[ \lambda_{i} \right]=0.075.$$

- $E\left[ H_{G}^{'} \right]=\frac{E\left[ \frac{N_{i}}{c} \right]}{E\left[ D_{i} \right]}$
  - See that $E\left[ \frac{\sum_{i}^{G} \left[ \frac{N_{i}}{c} \right]}{\sum_{i}^{G} D_{i}} \right]=\frac{\frac{1}{G}E\left[ \frac{N_{i}}{c} \right]}{\frac{1}{G}E[D_{i}]}=\frac{E\left[ \frac{N_{i}}{c} \right]}{E\left[ D_{i} \right]}\to^{c}\frac{\int_{0}^{\infty} \lambda\frac{1-e^{-120\cdot\gamma\cdot0.0817\cdot\lambda}}{1-e^{-\gamma\cdot0.0817\cdot\lambda}}f_{\lambda}\left( \lambda\right)d\lambda}{\int_{0}^{\infty} \frac{1-e^{-120\cdot\gamma\cdot0.0817\cdot\lambda}}{1-e^{-\gamma\cdot0.0817\cdot\lambda}}f_{\lambda}\left( \lambda\right)d\lambda}.$

It’s worth remembering $f_{\lambda}\left( \lambda\right)$ is given in closed form by the lognormal distribution. Using numeric integration, we present estimates in table A1.

# Appendix B

B.1 Hospital Readmissions Reduction Program (HRRP): Background and Clinical Validity of the 30-Day Risk Standardized Readmission Rate

The problem of comparing patient outcomes across different hospitals has been discussed by statisticians and epidemiologists since 1860 (Vandenbroucke & Vandenbroucke-Grauls, 1988). Health economists have used econometric techniques to develop measures of the impact of hospital differences on patient outcomes (Doyle et al., 2015, 2019).

The CMS uses various hospital-performance measures for its reporting and reform initiatives. It publicly reports its Hospital-Wide All-Cause Readmission (HWR) measure for eligible acute care hospitals as part of its Hospital Inpatient Quality Reporting (IQR) Program. The IQR program was mandated by Congressional legislation, which required hospitals to submit “data that relate to the quality of care furnished by the hospital" (U.S. Congress, 2003).

CMS calculates payment reduction using a formula that penalizes hospitals for what are termed excess readmissions. Specifically, the payment reduction formula relies on risk-standardized readmission measures for six conditions or procedures. These readmission measures are calculated for each of the six conditions/procedures, meaning that the index admission must have a discharge diagnosis corresponding to one of these six conditions. However, the readmission itself can be for any unplanned cause. These readmission measures are the only performance measures that are used to penalize hospitals under the HRRP.

Other CMS value-based programs reward or penalize hospitals based on different performance measures. For instance, the Hospital Value-Based Purchasing (VBP) Program uses outcomes such as mortality and spending to reward hospitals with incentive payments for the quality of care they provide.

The readmission measures used in both the IQR program and HRRP are based on 30-day unplanned all-cause readmissions. These measures are created using the same risk-standardization methodology. We use the HWR measure as our running example in the main text to refer to the HRRP readmission measures used by the CMS.^[[3]](#footnote-3)^

The 2012 original methodology report for the HWR measure argues that 30 days is a clinically reasonable timeframe for defining a hospital quality measure (Horwitz et al., 2012). The reasoning for this argument is based, in part, on randomized controlled trials that broadly study the effect of discharge planning on readmissions.^[[4]](#footnote-4)^ However, discharge planning seems to be related more to the nature of transitional care and a patient’s compliance to care instructions in an outpatient setting post-discharge than to the hospital’s quality of care during the patient’s inpatient stay. The available studies do not show why the 30-day timeframe makes readmission rates a suitable measure of hospital performance during the inpatient stay. Indeed, research indicates that early readmissions are generally more preventable, while readmissions occurring after seven days within the 30-day post-discharge period tend to be less related to factors during the index hospitalization (Graham et al., 2018).

In so far as the HRRP aims to link payment to quality of care, it is unclear why the payment reduction formula should factor in only readmissions and not mortality rates, which the CMS uses in its Hospital VBP program. Generally, a hospitalization is included in the calculation of the HWR as an index admission if the patient was alive upon discharge and continuously enrolled for 30 days in fee-for-service (FFS) Medicare Part A after discharge. However, patients who die within 30 days after discharge and thus have less than 30 days post-discharge enrollment in Medicare FFS are also eligible for inclusion (DeBuhr et al., 2024). This means that while an unplanned readmission before death is captured in the HWR, death within 30 days of discharge from the index admission without a readmission counts as a “zero readmission". The death does, however, count towards a 30-day mortality rate. This death could be linked to the quality of care received in the hospital during the index admission.

A clinician may not prioritize preventing an unplanned readmission if it helps reduce the risk of death. Further, the extent to which a readmission indicates poor quality of care varies based on the condition. Higher 30-day readmission rates could even suggest good quality of care for some of the six conditions/procedures for which risk-standardized readmission measures are calculated in the HRRP. For example, heart failure mortality rates are negatively associated with readmissions (Gorodeski et al., 2010). Thus, a clinician's treatment to reduce 30-day mortality risk among patients hospitalized for a specific disease may not align with the goal of reducing 30-day readmission rates. However, this clinician’s treatment would align with the Hospital VBP program because this program uses 30-day mortality (and not readmission) as a clinical outcome.

Both the Hospital VBP program and HRRP apply to the majority of acute care hospitals that receive payment under the Inpatient Prospective Payment System (IPPS). Future research should study how to jointly assess the effect of a bundle of incentives from CMS pay-for-performance programs that use different clinical outcomes to evaluate providers’ behavior and hospital practices.^[[5]](#footnote-5)^ There is likely heterogeneity in how hospitals respond to these various programs. Incentives to improve certain mortality measures in the Hospital VBP program vary across hospitals, with some hospitals having no incentives to improve on specific measures (Norton et al., 2023). It is unclear how clinicians respond to these bundles of incentives when treating diseases or conditions where mortality is negatively associated with readmission.

Some providers have expressed concerns about the proliferation and development of CMS quality measures (Jacobs et al., 2023; Talutis et al., 2019). Clinical researchers have argued that linking quality measures to reimbursement could discourage providers from serving vulnerable populations (Maddox, 2018). This suggests that providers are influenced by various clinical and financial incentives. Indeed, following the implementation of the inpatient prospective payment system, hospitals sought to reduce the average length of stay for Medicare beneficiaries, as the system reimbursed hospitals a fixed amount per hospitalization rather than based on the duration of the stay (Barnett et al., 2017).

B.2 HRRP Effectiveness and Incentives

The CMS, through the HRRP program, aims to reduce all-cause readmissions broadly—the payment reduction formula used to penalize hospitals aggregates condition-specific standardized readmission ratios for six conditions/procedures. The program does not distinguish between condition-specific challenges to preventing readmissions.

Some policymakers recommend that CMS move away from condition-specific measures and use the HWR measure instead because it is hospital-wide (Zuckerman et al., 2017). However, this could further incentivize hospitals to reduce readmissions more than mortality (Abdul-Aziz et al., 2017; Jha, 2018), depending on the incentives to increase survival from the Hospital VBP program. By penalizing readmissions that help clinicians lower mortality rates among patients with a specific chronic disease, the HRRP may be misaligning hospital and clinician incentives.

Understanding the effectiveness of payment reductions in preventing readmissions is further complicated by the unclear impact of the public reporting of the CMS readmission rates on the quality of healthcare provided by hospitals. According to theoretical and empirical health economics research, the disclosure of health care quality information is likely to affect the behavior of physicians and hospitals (Dranove & Jin, 2010). This literature is divided on whether disclosure would result in better or poorer quality of health care. Physicians and hospitals could try to “game" the reported quality and negatively affect patient outcomes by avoiding sick patients (Dranove et al., 2003). But public readmission rates could also help regulate quality by incentivizing hospitals to produce quality at the national average readmission rate (Vatter, 2024).

Himmelstein and Woolhandler (2015) argue that, in response to the HRRP payment reductions for excessive readmissions, hospitals appear to be treating recently discharged patients in emergency departments without readmitting them. They also seem to be treating these patients in inpatient units while classifying them as being in observation status, preventing them from appearing in inpatient statistics (Sabbatini et al. 2022). If true, this practice would leave patients worse off financially and could also affect their health outcomes through decreased quality of care.

The Medicare Payment Advisory Commission (MedPAC), on the other hand, is dismissive of Himmelstein and Woolhandler (2015). Their report (MedPAC, 2018) notes that readmission rates declined between 2010 and 2016, following the implementation of the HRRP, without increasing risk-adjusted mortality. Other clinical research has also argued that these readmission trends are likely due to program incentives (Zuckerman et al., 2016).

Some economists have contributed to the debate, arguing that the HRRP’s impact on reducing readmissions is either negligible or smaller than reported in certain clinical research articles (Ody et al., 2019). A significant share of the reduction in readmissions after the implementation of the HRRP may be due to other factors, such as hospitals increasing the coded severity of patients’ illness (Ibrahim et al., 2018). This is because the recorded diagnoses used for risk standardization reflect not only the patient’s underlying health status but also the provider’s tendency to ‘upcode’ when making a diagnosis (Finkelstein et al., 2017). On the other hand, Gupta (2021) finds empirical evidence that the program succeeded in motivating better quality of care by hospitals, despite readmissions reductions due to manipulation.

In sum, the effectiveness of the HRRP in reducing readmissions remains uncertain, indicating the need for further research.

# Appendix C

Consider two hospitals indexed by $1$ and $2.$ Each hospital serves one patient. Let $\gamma_{i}=\hat{\beta}Z_{1i}>0$ denote the estimated coefficient vector multiplied by the covariate vector for patient 1 at hospital $i$.

**Proposition C1**. *If* $\hat{\alpha_{2}}<\hat{\alpha_{1}}<\mu$*, then, for a given* $\gamma_{1}$*, there exists* $\gamma_{2}>\gamma_{1}$ *such that* $\hat{s}_{2}>\hat{s}_{1}$*.*

*Proof.* We will show $\exists\gamma_{2}$ such that $\hat{s}_{1}-\hat{s}_{2}<0$.

Write:

$$\hat{s}_{1}-\hat{s}_{2}=\frac{1+e^{-\left( \mu+\gamma_{1} \right)}}{1+e^{-\left( \hat{\alpha_{1}}+\gamma_{1} \right)}}-\frac{1+e^{-\left( \mu+\gamma_{2} \right)}}{1+e^{-\left( \hat{\alpha_{2}}+\gamma_{2} \right)}}.$$

Rewriting,

$$\hat{s}_{1}-\hat{s}_{2}=\frac{\left( 1+e^{-\left( \mu+\gamma_{1} \right)} \right)\left( 1+e^{-\left( \hat{\alpha_{2}}+\gamma_{2} \right)} \right)-\left( 1+e^{-\left( \mu+\gamma_{2} \right)} \right)\left( 1+e^{-\left( \hat{\alpha_{1}}+\gamma_{1} \right)} \right)}{\left( 1+e^{-\left( \hat{\alpha_{1}}+\gamma_{1} \right)} \right)\left( 1+e^{-\hat{(\alpha_{2}}+\gamma_{2})} \right)}.$$

The denominator is always positive. Thus, we denote the numerator $N$ and show $N<0$. Now,

$$N=\left( e^{-\left( \hat{\alpha_{2}}+\gamma_{2} \right)}+e^{-\left( \mu+\gamma_{1} \right)}+e^{-\left( \mu+\gamma_{1}+\hat{\alpha_{2}}+\gamma_{2} \right)} \right)-\left( e^{-\left( \hat{\alpha_{1}}+\gamma_{1} \right)}+e^{-\left( \mu+\gamma_{2} \right)}+e^{-\left( \mu+\gamma_{2}+\hat{\alpha_{1}}+\gamma_{1} \right)} \right).$$

Let

$$\begin{matrix} A & =\left( e^{-\left( \hat{\alpha_{2}}+\gamma_{2} \right)}-e^{-\left( \hat{\alpha_{1}}+\gamma_{1} \right)} \right) \\ B & =\left( e^{-\left( \mu+\gamma_{1} \right)}-e^{-\left( \mu+\gamma_{2} \right)} \right) \\ C & =\left( e^{-\left( \mu+\gamma_{1}+\hat{\alpha_{2}}+\gamma_{2} \right)}-e^{-\left( \mu+\gamma_{2}+\hat{\alpha_{1}}+\gamma_{1} \right)} \right). \end{matrix}$$

We can write $N=A+B+C$. For $A<0,$ we require $\hat{\alpha_{2}}+\gamma_{2} > \hat{\alpha_{1}}+\gamma_{1}$. Since we are proving the existence of such a $\gamma_{2},$ we can choose $\gamma_{2}$to be large enough such that $\gamma_{2}>\gamma_{1}+ \hat{\alpha_{1}}-\hat{\alpha_{2}}$. Under this condition, $A<0.$

Furthermore, because $\gamma_{2}>\gamma_{1},$ it follows that $B>0$. We choose $\gamma_{2}$to be sufficiently large allowing us to ensure $A<-\left( B+C \right).$

Having $\mu>\hat{\alpha_{1}},\hat{\alpha_{2}}$ allows $A+B<0.$ Further, $C>0$ always since $\hat{\alpha_{1}}>\hat{\alpha_{2}}$. Then, we have that $N<0.$ ◻

**Proposition C2**. *If* $\hat{\alpha_{1}}>\hat{\alpha_{2}}>\mu$*, then, for a given* $\gamma_{2}$*, there exists* $\gamma_{1}>\gamma_{2}$ *such that* $\hat{s}_{2}>\hat{s}_{1}$*.*

*Proof.* We will show $\exists\gamma_{1}$ such that $\hat{s}_{1}-\hat{s}_{2}<0$.

Write:

$$\hat{s}_{1}-\hat{s}_{2}=\frac{1+e^{-\left( \mu+\gamma_{1} \right)}}{1+e^{-\left( \hat{\alpha_{1}}+\gamma_{1} \right)}}-\frac{1+e^{-\left( \mu+\gamma_{2} \right)}}{1+e^{-\left( \hat{\alpha_{2}}+\gamma_{2} \right)}}.$$

Rewriting,

$$\hat{s}_{1}-\hat{s}_{2}=\frac{\left( 1+e^{-\left( \mu+\gamma_{1} \right)} \right)\left( 1+e^{-\left( \hat{\alpha_{2}}+\gamma_{2} \right)} \right)-\left( 1+e^{-\left( \mu+\gamma_{2} \right)} \right)\left( 1+e^{-\left( \hat{\alpha_{1}}+\gamma_{1} \right)} \right)}{\left( 1+e^{-\left( \hat{\alpha_{1}}+\gamma_{1} \right)} \right)\left( 1+e^{-(\hat{\alpha_{2}}+\gamma_{2})} \right)}.$$

The denominator is always positive. Thus, we denote the numerator $N$ and show $N<0$. Now,

$$N=\left( e^{-\left( \hat{\alpha_{2}}+\gamma_{2} \right)}+e^{-\left( \mu+\gamma_{1} \right)}+e^{-\left( \mu+\gamma_{1}+\hat{\alpha_{2}}+\gamma_{2} \right)} \right)-\left( e^{-\left( \hat{\alpha_{1}}+\gamma_{1} \right)}+e^{-\left( \mu+\gamma_{2} \right)}+e^{-\left( \mu+\gamma_{2}+\hat{\alpha_{1}}+\gamma_{1} \right)} \right).$$

Let

$$\begin{matrix} A & =\left( e^{-\left( \hat{\alpha_{2}}+\gamma_{2} \right)}-e^{-\left( \hat{\alpha_{1}}+\gamma_{1} \right)} \right) \\ B & =\left( e^{-\left( \mu+\gamma_{1} \right)}-e^{-\left( \mu+\gamma_{2} \right)} \right) \\ C & =\left( e^{-\left( \mu+\gamma_{1}+\hat{\alpha_{2}}+\gamma_{2} \right)}-e^{-\left( \mu+\gamma_{2}+\hat{\alpha_{1}}+\gamma_{1} \right)} \right). \end{matrix}$$

We can write $N=A+B+C$. For $B<0,$ we require $\mu+\gamma_{1} > \mu+\gamma_{2}$, which implies $\gamma_{1} >\gamma_{2}$. Since we are proving the existence of such a $\gamma_{1},$ we can choose $\gamma_{1}$to be large enough such that $\gamma_{1}>\gamma_{2}$. Under this condition, $B<0.$

Furthermore, because $\hat{\alpha_{1}}>\hat{\alpha_{2}}$ and $\gamma_{1}>\gamma_{2},$ it follows that $A>0.$We choose $\gamma_{1}$to be sufficiently large allowing us to ensure $B<-\left( A+C \right).$

Having $\mu<\hat{\alpha_{1}},\hat{\alpha_{2}}$ allows $B+A<0.$ Further, $C>0$ always since $\hat{\alpha_{1}}>\hat{\alpha_{2}}$. Then, we have that $N<0.$ ◻

# References

Abdul-Aziz, A. A., Hayward, R. A., Aaronson, K. D., & Hummel, S. L. (2017). Association Between Medicare Hospital Readmission Penalties and 30-Day Combined Excess Readmission and Mortality. *JAMA Cardiology*, *2*(2), 200. <https://doi.org/10.1001/jamacardio.2016.3704>

Barnett, M. L., Grabowski, D. C., & Mehrotra, A. (2017). Home-to-Home Time—Measuring What Matters to Patients and Payers. *New England Journal of Medicine*, *377*(1), 4–6. <https://doi.org/10.1056/NEJMp1703423>

Centers for Medicare & Medicaid Services (CMS). (2023). *Hybrid Hospital-wide Risk-Standardized Mortality Measure with Electronic Health Record Extracted Risk Factors Methodology Report.* Retrieved from <https://www.cms.gov/files/document/hybrid-hospital-wide-all-condition-all-procedure-risk-standardized-mortality-measure-electronic.pdf>

Centers for Medicare & Medicaid Services (CMS). (2024). *Readmission Measures Methodology*. <https://qualitynet.cms.gov/inpatient/measures/readmission/methodology>

Coleman, E. A., Smith, J. D., Frank, J. C., Min, S.-J., Parry, C., & Kramer, A. M. (2004). Preparing patients and caregivers to participate in care delivered across settings: The Care Transitions Intervention. *Journal of the American Geriatrics Society*, *52*(11), 1817–1825. <https://doi.org/10.1111/j.1532-5415.2004.52504.x>

Courtney, M., Edwards, H., Chang, A., Parker, A., Finlayson, K., & Hamilton, K. (2009). Fewer emergency readmissions and better quality of life for older adults at risk of hospital readmission: A randomized controlled trial to determine the effectiveness of a 24-week exercise and telephone follow-up program. *Journal of the American Geriatrics Society*, *57*(3), 395–402. <https://doi.org/10.1111/j.1532-5415.2009.02138.x>

Doyle, J. J., Graves, J. A., & Gruber, J. (2019). Evaluating Measures of Hospital Quality: Evidence from Ambulance Referral Patterns. *The Review of Economics and Statistics*, *101*(5), 841–852. <https://doi.org/10.1162/rest_a_00804>

Doyle, J. J., Graves, J. A., Gruber, J., & Kleiner, S. A. (2015). Measuring Returns to Hospital Care: Evidence from Ambulance Referral Patterns. *Journal of Political Economy*, *123*(1), 170–214. <https://doi.org/10.1086/677756>

Dranove, D., & Jin, G. Z. (2010). Quality Disclosure and Certification: Theory and Practice. *Journal of Economic Literature*, *48*(4), 935–963. <https://doi.org/10.1257/jel.48.4.935>

Dranove, D., Kessler, D., McClellan, M., & Satterthwaite, M. (2003). Is More Information Better? The Effects of “Report Cards” on Health Care Providers. *Journal of Political Economy*, *111*(3), 555–588. <https://doi.org/10.1086/374180>

Finkelstein, A., Gentzkow, M., Hull, P., & Williams, H. (2017). Adjusting Risk Adjustment—Accounting for Variation in Diagnostic Intensity. *New England Journal of Medicine*, *376*(7), 608–610. <https://doi.org/10.1056/NEJMp1613238>

Garåsen, H., Windspoll, R., & Johnsen, R. (2007). Intermediate care at a community hospital as an alternative to prolonged general hospital care for elderly patients: A randomised controlled trial. *BMC Public Health*, *7*, 68. <https://doi.org/10.1186/1471-2458-7-68>

Gorodeski, E. Z., Starling, R. C., & Blackstone, E. H. (2010). Are All Readmissions Bad Readmissions? *New England Journal of Medicine*, *363*(3), 297–298. <https://doi.org/10.1056/NEJMc1001882>

Graham, K. L., Auerbach, A. D., Schnipper, J. L., Flanders, S. A., Kim, C. S., Robinson, E. J., Ruhnke, G. W., Thomas, L. R., Kripalani, S., Vasilevskis, E. E., Fletcher, G. S., Sehgal, N. J., Lindenauer, P. K., Williams, M. V., Metlay, J. P., Davis, R. B., Yang, J., Marcantonio, E. R., & Herzig, S. J. (2018). Preventability of Early Versus Late Hospital Readmissions in a National Cohort of General Medicine Patients. *Annals of Internal Medicine*, *168*(11), 766–774. <https://doi.org/10.7326/M17-1724>

Gupta, A. (2021). Impacts of Performance Pay for Hospitals. *The American Economic Review*, *111*(4), 1241–1283. JSTOR. <https://doi.org/10.1257/aer.20171825>

Himmelstein, D., & Woolhandler, S. (2015). *Quality Improvement: ‘Become Good At Cheating And You Never Need To Become Good At Anything Else.’* <https://doi.org/10.1377/hblog20150827.050132>

Ibrahim, A. M., Dimick, J. B., Sinha, S. S., Hollingsworth, J. M., Nuliyalu, U., & Ryan, A. M. (2018). Association of Coded Severity With Readmission Reduction After the Hospital Readmissions Reduction Program. *JAMA Internal Medicine*, *178*(2), 290–292. <https://doi.org/10.1001/jamainternmed.2017.6148>

Jack, B. W., Chetty, V. K., Anthony, D., Greenwald, J. L., Sanchez, G. M., Johnson, A. E., Forsythe, S. R., O’Donnell, J. K., Paasche-Orlow, M. K., Manasseh, C., Martin, S., & Culpepper, L. (2009). A Reengineered Hospital Discharge Program to Decrease Rehospitalization: A Randomized Trial. *Annals of Internal Medicine*, *150*(3), 178. <https://doi.org/10.7326/0003-4819-150-3-200902030-00007>

Jacobs, D. B., Schreiber, M., Seshamani, M., Tsai, D., Fowler, E., & Fleisher, L. A. (2023). Aligning Quality Measures across CMS — The Universal Foundation. *New England Journal of Medicine*, *388*(9), 776–779. <https://doi.org/10.1056/NEJMp2215539>

Jha, A. K. (2018). To Fix the Hospital Readmissions Program, Prioritize What Matters. *JAMA*, *319*(5), 431. <https://doi.org/10.1001/jama.2017.21623>

Koehler, B. E., Richter, K. M., Youngblood, L., Cohen, B. A., Prengler, I. D., Cheng, D., & Masica, A. L. (2009). Reduction of 30-day postdischarge hospital readmission or emergency department (ED) visit rates in high-risk elderly medical patients through delivery of a targeted care bundle. *Journal of Hospital Medicine*, *4*(4), 211–218. <https://doi.org/10.1002/jhm.427>

Maddox, K. E. J. (2018). Financial Incentives and Vulnerable Populations—Will Alternative Payment Models Help or Hurt? *New England Journal of Medicine*, *378*(11), 977–979. <https://doi.org/10.1056/NEJMp1715455>

Medicare Payment Advisory Committee (MedPAC). (2018). *Mandated report: The effects of the Hospital Readmissions Reduction Program*. [ttps://www.medpac.gov/wp-content/uploads/import_data/scrape_files/docs/default-source/reports/jun18_ch1_medpacreport_rev_nov2019_v2_note_sec.pdf](https://doi.org/ttps:/www.medpac.gov/wp-content/uploads/import_data/scrape_files/docs/default-source/reports/jun18_ch1_medpacreport_rev_nov2019_v2_note_sec.pdf)

Metcalfe, C., Thompson, S. G., Cowie, M. R., & Sharples, L. D. (2003). The use of hospital admission data as a measure of outcome in clinical studies of heart failure. *European Heart Journal*, *24*(1), 105–112. <https://doi.org/10.1016/S0195-668X(02)00384-6>

Mistiaen, P., Francke, A. L., & Poot, E. (2007). Interventions aimed at reducing problems in adult patients discharged from hospital to home: A systematic meta-review. *BMC Health Services Research*, *7*, 47. <https://doi.org/10.1186/1472-6963-7-47>

Naylor, M., Brooten, D., Jones, R., Lavizzo-Mourey, R., Mezey, M., & Pauly, M. (1994). Comprehensive discharge planning for the hospitalized elderly: A randomized clinical trial. *Annals of Internal Medicine*, *120*(12), 999–1006. <https://doi.org/10.7326/0003-4819-120-12-199406150-00005>

Naylor, M. D., Brooten, D., Campbell, R., Jacobsen, B. S., Mezey, M. D., Pauly, M. V., & Schwartz, J. S. (1999). Comprehensive Discharge Planning and Home Follow-up of Hospitalized Elders: A Randomized Clinical Trial. *JAMA*, *281*(7), 613–620. <https://doi.org/10.1001/jama.281.7.613>

Norton, E. C., Lawton, E. J., & Li, J. (2023). Moneyball in Medicare: Heterogeneous Treatment Effects. *American Journal of Health Economics*, *9*(1), 96–126. <https://doi.org/10.1086/721707>

Ody, C., Msall, L., Dafny, L. S., Grabowski, D. C., & Cutler, D. M. (2019). Decreases In Readmissions Credited To Medicare’s Program To Reduce Hospital Readmissions Have Been Overstated. *Health Affairs*, *38*(1), 36–43. <https://doi.org/10.1377/hlthaff.2018.05178>

Pope, D. G. (2009). Reacting to rankings: Evidence from “America’s Best Hospitals.” *Journal of Health Economics*, *28*(6), 1154–1165. <https://doi.org/10.1016/j.jhealeco.2009.08.006>

Sabbatini, A. K., Joynt-Maddox, K. E., Liao, J. M., Basu, A., Parrish, C., Kreuter, W., & Wright, B. (2022). Accounting for the Growth of Observation Stays in the Assessment of Medicare’s Hospital Readmissions Reduction Program. *JAMA Network Open*, *5*(11), e2242587. <https://doi.org/10.1001/jamanetworkopen.2022.42587>

Stauffer, B. D., Fullerton, C., Fleming, N., Ogola, G., Herrin, J., Stafford, P. M., & Ballard, D. J. (2011). Effectiveness and cost of a transitional care program for heart failure: A prospective study with concurrent controls. *Archives of Internal Medicine*, *171*(14), 1238–1243. <https://doi.org/10.1001/archinternmed.2011.274>

Talutis, S. D., Chen, Q., Wang, N., & Rosen, A. K. (2019). Comparison of Risk-Standardized Readmission Rates of Surgical Patients at Safety-Net and Non–Safety-Net Hospitals Using Agency for Healthcare Research and Quality and American Hospital Association Data. *JAMA Surgery*, *154*(5), 391. <https://doi.org/10.1001/jamasurg.2018.5242>

U.S. Congress. (2003). Medicare Prescription Drug, Improvement, and Modernization Act of 2003 (Public Law 108-173). Retrieved from <https://www.congress.gov/108/plaws/publ173/PLAW-108publ173.pdf>

Vandenbroucke, J. P., & Vandenbroucke-Grauls, C. M. (1988). A Note on the History of the Calculation of Hospital Statistics. *American Journal of Epidemiology*, *127*(4), 699–702. <https://doi.org/10.1093/oxfordjournals.aje.a114850>

Vatter, B. (2024). *Quality Disclosure and Regulation: Scoring Design in Medicare Advantage* (SSRN Scholarly Paper 4250361). <https://doi.org/10.2139/ssrn.4250361>

Voss, R., Gardner, R., Baier, R., Butterfield, K., Lehrman, S., & Gravenstein, S. (2011). The Care Transitions Intervention: Translating From Efficacy to Effectiveness. *Archives of Internal Medicine*, *171*(14), 1232–1237. <https://doi.org/10.1001/archinternmed.2011.278>

Walraven, C. van, Seth, R., Austin, P. C., & Laupacis, A. (2002). Effect of Discharge Summary Availability During Post-discharge Visits on Hospital Readmission. *Journal of General Internal Medicine*, *17*(3), 186. <https://doi.org/10.1046/j.1525-1497.2002.10741.x>

Walsh, E. G., Freiman, M., Haber, S., Bragg, A., Ouslander, J., & Wiener, J. M. (2010). Cost Drivers for Dually Eligible Beneficiaries: Potentially Avoidable Hospitalizations from Nursing Facility, Skilled Nursing Facility, and Home and Community-Based Services Waiver Programs. Retrieved from <https://www.cms.gov/research-statistics-data-and-systems/statistics-trends-and-reports/reports/downloads/costdriverstask2.pdf>

Weiss, M., Yakusheva, O., & Bobay, K. (2010). Nurse and patient perceptions of discharge readiness in relation to postdischarge utilization. *Medical Care*, *48*(5), 482–486. <https://doi.org/10.1097/MLR.0b013e3181d5feae>

Zuckerman, R. B., Joynt Maddox, K. E., Sheingold, S. H., Chen, L. M., & Epstein, A. M. (2017). Effect of a Hospital-wide Measure on the Readmissions Reduction Program. *New England Journal of Medicine*, *377*(16), 1551–1558. <https://doi.org/10.1056/NEJMsa1701791>

1. See Table 21 (Walsh et al., 2010). The number of patient-years is not listed, nevertheless we can find the answer via the formula:

   $\frac{958,837}{382,846}\cdot\frac{360 \mathrm{hospitalizations}}{1,000 patient-years}\cdot\frac{\frac{1}{1000} patient-years}{\frac{1}{1000} patient-years}=0.90\frac{\mathrm{hospitalizations}}{1 patient-year}=0.075 \frac{\mathrm{hospitalizations}}{1 patient-month}$ [↑](#footnote-ref-1)
2. 8.17% is stated in the introduction. The same source also provides the number 6.30% as an alternative (see table G.2). The particulars of the way multiple hospitalizations in a 30-day period may interact together is not examined outside of the multiplicative form $\left( 1-\frac{1}{c} \gamma\cdot0.0817 \right)^{n_{it}}$. For example, the specified formula treats the censoring impact if $n_{it}=1$ or $n_{it}=2$ equally (though it’s rare in our setup for multiple hospitalizations to occur in a given month). For cohort studies, other issues like time-varying incidence rates may drive the divergence between $H_{G}^{'}$ and $H_{G}$ more than the dependent censoring mechanism examined here (Metcalfe et al., 2003). [↑](#footnote-ref-2)
3. See (Centers for Medicare & Medicaid Services [CMS], 2024) to access methodology reports for the CMS readmission measures. [↑](#footnote-ref-3)
4. See (Coleman et al., 2004; Courtney et al., 2009; Garåsen et al., 2007; Jack et al., 2009; Koehler et al., 2009; Mistiaen et al., 2007; M. Naylor et al., 1994; M. D. Naylor et al., 1999; Stauffer et al., 2011; Voss et al., 2011; Walraven et al., 2002; Weiss et al., 2010) for RCT studies cited in the original report. [↑](#footnote-ref-4)
5. Health economists recognize that hospital quality measures are multidimensional and often controversial, which makes it difficult to measure quality (Doyle et al., 2019; Pope, 2009). While measures of hospital quality are widely debated, we could not find any paper that discusses how to account for interactions among various quality-improving policies and their associated incentives when measuring hospital quality. [↑](#footnote-ref-5)
