# Supplementary material for: Assessing the Estimands and Estimates of Hospitalization Rates in Health Economics and Clinical Medicine
Source: Health Econ. 2026 Jun 18;35(9):1397–405. doi: 10.1002/hec.70117 (PMC13428625; doi:10.1002/hec.70117)
Supplement: Supplementary file 2 — Supporting Information S2 [file HEC-35-1397-s002.docx]

# Appendix Figures


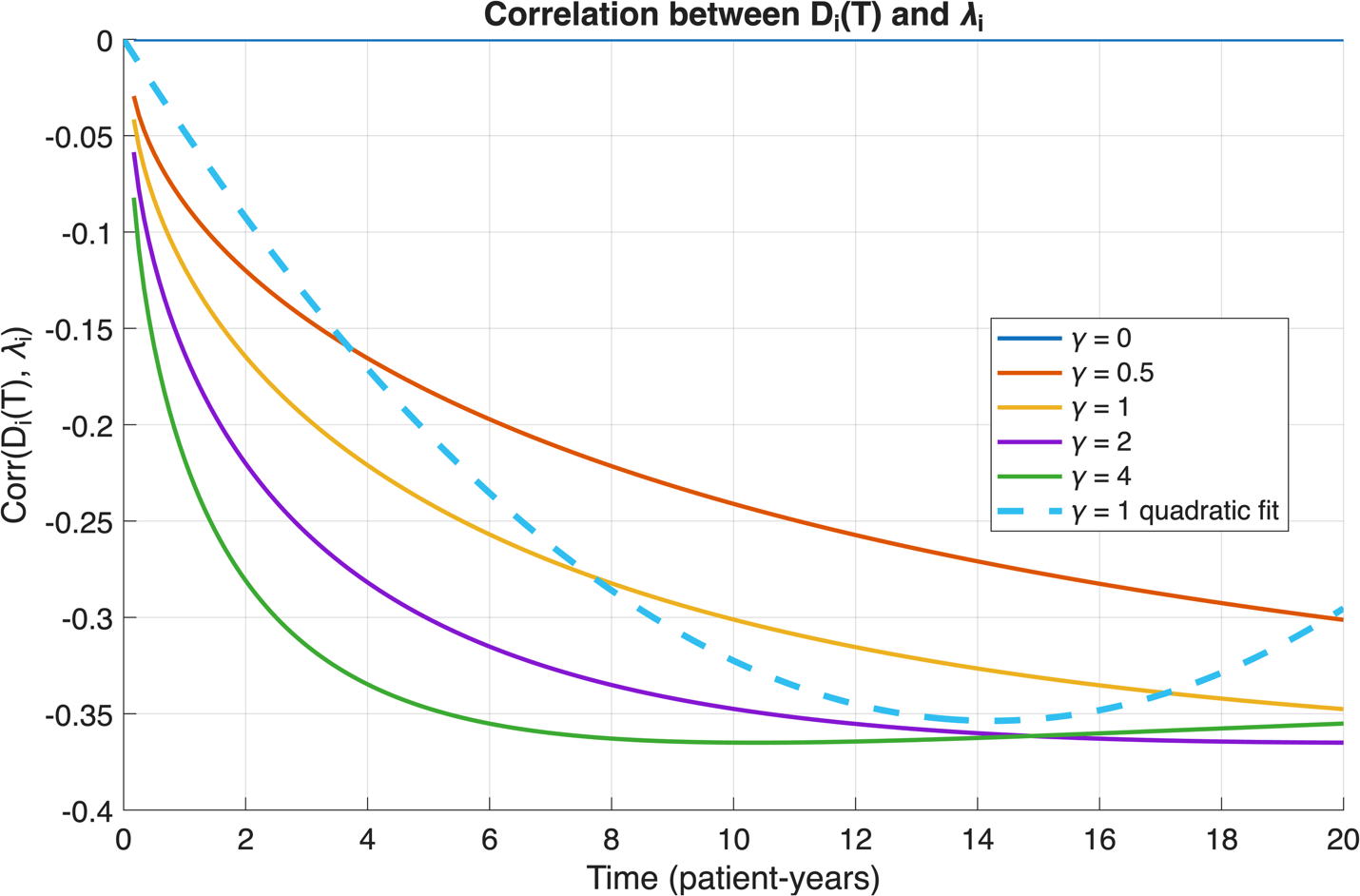


**Figure A1**: This figure presents the correlation between total follow-up time $D_{i}(T)$ and the underlying hospital admission rate $\lambda_{i}$over maximum follow-up duration in terms of patient-years ($T/12).$ The parameter $\gamma$ scales the baseline mortality rate of 8.17%. When $\gamma=0,$ there is no correlation. For large $T$, higher $\gamma$ values do not always yield stronger negative correlations, demonstrating that the relationship between the correlation and T is not necessarily monotonic.
