# Supplementary material for: Assessing the Estimands and Estimates of Hospitalization Rates in Health Economics and Clinical Medicine
Source: Health Econ. 2026 Jun 18;35(9):1397–405. doi: 10.1002/hec.70117 (PMC13428625; doi:10.1002/hec.70117)
Supplement: Supplementary file 3 — Supporting Information S3 [file HEC-35-1397-s001.docx]

# Appendix Tables

Table A1: Patient Outcomes Under Mortality Assumptions

| $\boldsymbol{\gamma}$ | $\boldsymbol{E[}\boldsymbol{D}_{\boldsymbol{i}}\boldsymbol{]}$ | $\boldsymbol{E}\left[ \boldsymbol{\lambda}_{\boldsymbol{i}}\boldsymbol{D}_{\boldsymbol{i}} \right]\boldsymbol{=E}\left[ \frac{\boldsymbol{N}_{\boldsymbol{i}}}{\boldsymbol{c}} \right]$ | $\boldsymbol{Corr(}\boldsymbol{\lambda}_{\boldsymbol{i}}\boldsymbol{,}\boldsymbol{D}_{\boldsymbol{i}}\boldsymbol{)}$ | $\boldsymbol{E}\left[ \boldsymbol{H}_{\boldsymbol{G}}^{\boldsymbol{'}} \right]$ | $\boldsymbol{E[}\boldsymbol{H}_{\boldsymbol{G}}\boldsymbol{]}$ |
| --- | --- | --- | --- | --- | --- |
| **0** | 120 months | 9 admissions | 0.00 | 0.075 $\frac{\mathrm{admissions}}{patient-month}$ | 0.075 $\frac{admissions}{patient-month}$ |
| **0.5** | 101.10 | 7.25 | -0.24 | 0.072 | 0.075 |
| **1** | 86.79 | 6.01 | -0.30 | 0.069 | 0.075 |
| **2** | 66.75 | 4.40 | -0.35 | 0.066 | 0.075 |
| **4** | 44.39 | 2.77 | -0.37 | 0.062 | 0.075 |

Notes: This table presents results for the impact of the 30-day post-hospitalization mortality rate scaling factor, $\gamma,$ on patient outcomes. Values are computed using a baseline mortality rate of 8.17%. The columns report the expected follow-up time in months, $E[D_{i}],$the expected number of hospital admissions, $E[{\lambda_{i}D}_{i}$], and the correlation between the rate, $\lambda_{i},$and total follow-up time, $D_{i}$. The table also compares the hospitalization rate estimator $H_{G}^{'}$against our proposed alternative estimator $H_{G}\boldsymbol{,}$scaled to admissions per patient-month.
